# Diffuse Nickel Hypersensitivity Reaction Post-cholecystectomy in a Young Female

**DOI:** 10.7759/cureus.17146

**Published:** 2021-08-13

**Authors:** Enkhmaa Luvsannyam, Arathi Jayaraman, Molly S Jain, Ravi P Jagani, Veronica Velez, Anushka S Mirji, Frederick Tiesenga, Juaquito Jorge

**Affiliations:** 1 Research, California Institute of Behavioral Neurosciences & Psychology, Fairfield, USA; 2 Surgery, Avalon University School of Medicine, Youngstown, USA; 3 Medicine, Xavier University School of Medicine, Oranjestad, ABW; 4 Medicine, Saint James School of Medicine, Park Ridge, USA; 5 Medicine, All Saints University College of Medicine, Kingstown, VCT; 6 Surgery, Metropolitan University College of Medicine, St. John's, ATG; 7 General Surgery, West Suburban Medical Center, Chicago, USA; 8 General and Vascular Surgery, West Suburban Hospital, Oak Park, USA

**Keywords:** nickel, foreign body, type iv hypersensitivity, abdominal pain, post cholecystectomy, case report

## Abstract

Nickel, a silvery-hard metallic element used in corrosion-resistant alloys, is widely used in the medical field. Nickel has aided in medical advancements; however, it has been known to cause hypersensitivity reactions. Retained foreign bodies due to surgical procedures may cause postoperative complications such as allergic reactions. This case involves a 30-year-old female presenting with non-specific symptoms involving multiple organ systems, notably with abdominal pain. Due to chronic symptoms, the patient was tested for metal allergies and diagnosed with hypersensitivity reactions to nickel surgical clips that were previously inserted during cholecystectomy. Subsequently, the patient had surgical removal of the foreign bodies, which led to significant improvement of her symptoms immediately. This case demonstrates a delayed hypersensitivity reaction to a foreign body involving multiple body systems and vague symptoms making the diagnosis challenging. It is important to carefully evaluate the patient's past medical history including history of any allergies. It also brings attention to the necessity of performing metal skin patch tests preoperatively for individuals with a history of any type of allergies.

## Introduction

Nickel is a common metal used in different surgical equipment such as surgical clips, forceps, clamps, scissors, holders, and implants [1]. Nickel has several advantages such as being economical to manufacture, has good tensile strength, and is resistant to deterioration and high temperature [1]. Despite its advantages, the general population has developed allergic reactions to nickel commonly. In fact, the prevalence of nickel allergy is approximately 17% in women and 3% in men [2].

Nickel allergy can present as a wide array of syndromes such as contact dermatitis in type IV hypersensitivity reaction, lichen planus, dyshidrotic eczema, pustulosis palmoplantaris, and burning mouth syndrome [3]. Physical examination may reveal an acute phase of the reaction with erythema, induration, vesicles, bullae, scaling plaques, and edema. Chronic reactions can lead to dryness, hyperkeratosis, pruritus, scaling, lichenification, hyperpigmentation, and fissuring [4]. When a patient is allergic to nickel and exposed to implanted devices with nickel, it may lead to systemic reactions [4]. Nickel allergy can become quite a nuisance for the patient, especially in chronic cases. Baumann et al. report a case series in which multiple patients experience hypersensitivity to nickel-based orthopedic implants [5]. They concluded that the implants lead to hypersensitivity reactions that may result in many therapeutic and diagnostic challenges [5].

Nickel allergy is a chronic condition for a lifetime. An individual should avoid any form of nickel contact to their body, reducing the possibility of developing any reactions. The initial step of management is the removal of the offending agent. Some other therapeutic options available for allergic reactions include steroids, calcineurin inhibitors, disulfiram, and binding agents [6].

This report describes a case of retained nickel surgical clips during a cholecystectomy, which caused delayed non-specific systemic symptoms due to an unknown nickel allergy.

## Case presentation

This case involves a 30-year-old female who presented with non-specific symptoms involving multiple body systems due to nickel allergy for one year. The patient has no significant past medical history except for post-traumatic stress disorder. Her past surgical history includes tonsillectomy 15 years ago, left elbow surgery 10 years ago, and cholecystectomy a year ago. The patient is allergic to azithromycin, nonsteroidal anti-inflammatory drugs (NSAIDs), and nickel, which was unknown until the appearance of her symptoms post-cholecystectomy. Her father was diagnosed with high blood pressure; however, she denies any family history of allergic reactions.

**Figure 1:**
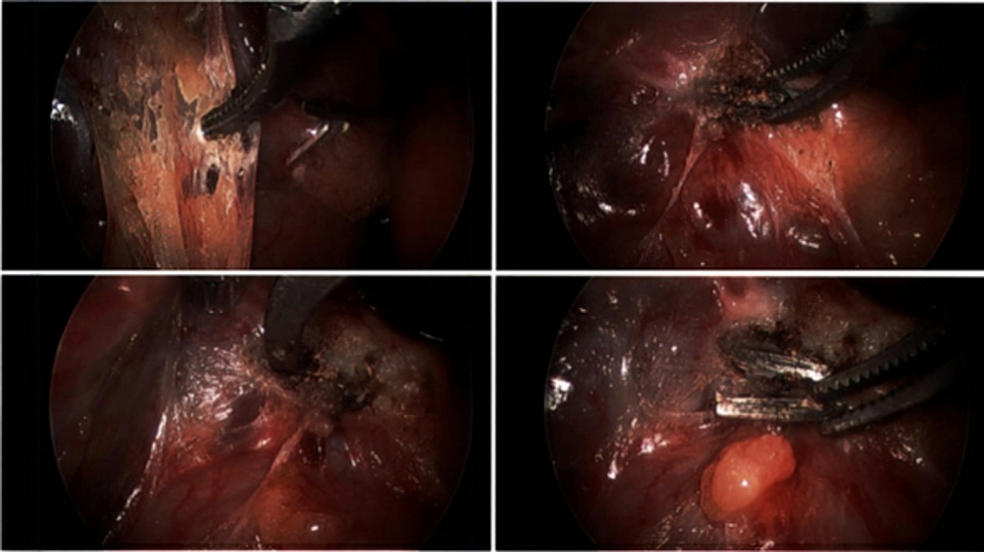
Diagnostic laparoscopy evaluation with fluoroscopic guidance was performed to locate the positioning of six metal surgical clips.

The patient had a gallbladder removal due to symptomatic cholelithiasis for eight months. Four weeks post-cholecystectomy, the patient initially presented to the clinic complaining of intermittent right upper quadrant (RUQ) pain that is worse than her preoperative pain. The patient describes the pain as progressive, burning, and pinching in quality. No alleviating or aggravating factors were reported and the pain was unrelated to food or physical activity. She denied fever, chills, nausea, vomiting, diarrhea, or other symptoms. On physical examination, her abdomen was soft, non-tender, non-distended with normal bowel sounds. Complete lab work showed no abnormalities. During the eight-week follow-up appointment, the patient was suffering from constant RUQ pain with no other significant symptoms. In the following month, the patient had intermittent tachycardia, dyspnea, and chest pressure for three weeks. Electrocardiogram (EKG) and upper endoscopy revealed no significant findings and her cardiac symptoms diminished in the following week. Eight months after the surgery, the patient came back to her primary care physician complaining of persistent RUQ pain and additional symptoms of flu-like illness. The patient had developed chronic headaches, neck pain and pressure, progressive dyspnea, right preauricular and postauricular lymphadenopathy, constant bloating, and severe constipation. She was also complaining of fatigue throughout the day and feeling heavy limbs. Her pain symptoms were slightly and temporarily relieved by Tylenol. The patient was not able to take NSAIDs such as ibuprofen due to her allergy. Another set of complete blood work was performed and was within normal limits. At this point, the patient was having an extremely difficult time due to her ongoing symptoms causing significant lifestyle discomfort and inability to concentrate on her work. Due to ongoing symptoms and normal lab results, the patient had a metal allergy test with a skin patch that turned out positive for a nickel allergy. Further confirmation with a complete orthopedic analysis-metal allergy test was performed and confirmed her nickel allergy. Considering the high possibility of nickel surgical clips retained during her cholecystectomy causing her symptoms, the decision was made to remove the surgical clips. A week after her visit, the patient had a diagnostic laparoscopy and removal of six metal surgical clips from her RUQ via fluoroscopic guidance (Figure 1).

**Figure 2:**
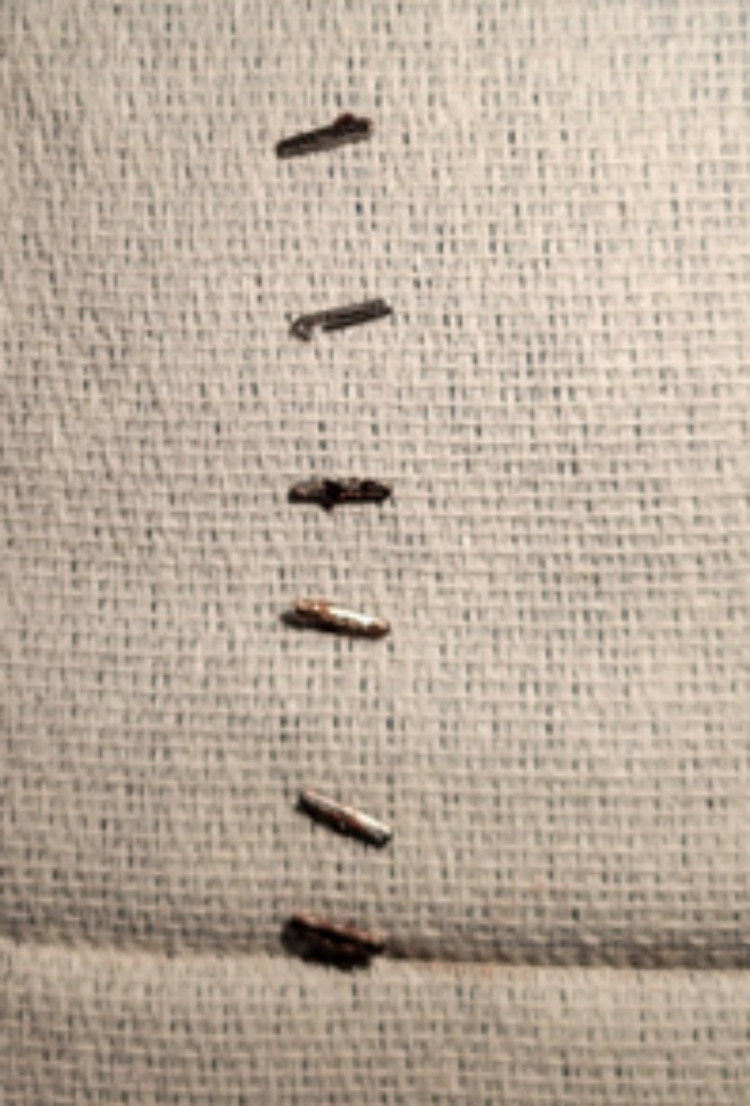
Six metal (nickel) surgical clips were identified and removed from the right upper quadrant with the assistance of fluoroscopic guidance.

The surgical pathology report specified “foreign body” and consists of six metallic surgical clips, each measuring 1.0 cm in length and 0.1 cm in diameter (Figure 2).

The patient tolerated the procedure well and there were no postoperative complications. One week postoperatively, her RUQ pain was diminished along with the other symptoms of headache, neck pain, lymphadenopathy, and bloating. The patient is recovering well presently. She has been recommended to do a close follow-up for few months to monitor her progress.

## Discussion

The body can have hypersensitivity reactions to two main antigens: self-antigens and environmental antigens. Autoimmune disorders occur when the body reacts to healthy self-antigens resulting from self-reactive T-cells and B-cells escaping destruction during maturation in the thymus [3]. Allergies occur when the body reacts more intensely than normal to harmless environmental antigens, such as nickel [3]. There are higher instances of metal allergies in individuals with autoimmune conditions such as systemic lupus erythematosus, rheumatoid arthritis, and Sjogren's syndrome [3]. Our patient does not have a history of autoimmune disorders. However, the patient has a history of allergies to common medications such as azithromycin and NSAIDs, which increases the risk of other allergic reactions such as nickel.

Four types of hypersensitivity reactions occur in people that are pre-sensitized [3]. Allergic reactions to metals are associated with type I and IV hypersensitivities [3]. Type I hypersensitivity reactions are immediate reactions that occur when pre-sensitization has produced immunoglobulin E (IgE) antibodies that cause mast and basophil cell degranulation upon re-exposure [3]. Type IV hypersensitivity reactions are delayed reactions where pre-sensitization produces sensitized cluster of differentiation 4 (CD4+) T-cells to release inflammatory cytokines and cluster of differentiation 8 (CD8+) T-cells to release cytotoxic cytokines [3].

The exact mechanism of nickel allergy is unknown [3]. Nickel ions release haptens which covalently bind to skin carrier proteins [4]. This activates keratinocyte cells to produce cytokines such as interleukin-1 beta (IL-1β) and tumor necrosis factor-alpha (TNF-α) as well as cytokines that activate both Langerhans and dendritic antigen-presenting cells (APCs) [4]. Nickel also attaches directly to the major histocompatibility complex (MHC) of Langerhans and dendritic cells [4]. The activated APCs then migrate to draining lymph nodes where they present the haptens to the naive CD4+ T-cells resulting in sensitized T-cells [4]. Reexposure to the nickel haptens causes the sensitized T-cells to enter the bloodstream and cause signs of hypersensitivity at 48 to 72 hours after reexposure [3]. Ingestion of nickel particles with a positive nickel patch test is called systemic nickel allergy syndrome (SNAS) and can result in contact dermatitis plus extracutaneous symptoms such as respiratory, gastrointestinal, and/or neurologic symptoms [7].

Systemic nickel allergy can also occur postoperatively after nickel-based surgical implants [8]. Of the orthopedic cases, 1% to 5% have reported cutaneous hypersensitivity reactions to implanted metals due to nickel ion leakage once implanted within the body [8]. In addition to implantable objects such as steel screws and metal surgical clips, operating equipment can also be a source of nickel haptens [8]. A study done in Hennepin County Medical Center (HCMC) in Minneapolis, Minnesota tested various operating room equipment for nickel release [1]. They found that nickel release from operating equipment is not common; however, it can vary at each operating theatre leading to hypersensitivity reactions [1].

For our patient, the diagnosis of systemic nickel allergy-causing contact dermatitis was aided by the metal skin patch test. Preoperative metal skin patch tests have been shown to be helpful for patients with a history of metal hypersensitivity prior to prosthetic device implantation [9]. However, a metal skin patch test is not routinely done preoperatively [10]. Our patient did not have a metal skin patch test done before her metal surgery clips implantation for the cholecystectomy due to no prior history of metal allergies.

The highlight of this case is delayed systemic hypersensitivity reaction due to metal foreign bodies. Since the patient has a history of allergic reactions to other agents, her symptoms may not be fully caused by nickel hypersensitivity initially. However, the positive skin patch test and alleviation of her symptoms after the removal of nickel surgical clips further confirm that nickel hypersensitivity had a major role in her clinical course. The limitation of this case pertains to the delayed clinical diagnosis. The nonspecific symptoms and widespread manifestation of the patient’s allergic reaction made the diagnosis challenging and caused her significant life distress. However, once the diagnosis was made and confirmed, the surgical intervention aided in immediate symptom relief. This limitation can be prevented with careful evaluation and appropriate metal allergy tests for a specific patient population.

## Conclusions

Delayed hypersensitivity reactions can present in multiple manners. It should be considered for patients presenting with nonspecific symptoms following surgical procedures that require the placement of nickel or any metal alloy in the body. Some specific symptoms such as abdominal pain and bloating following an abdominal surgery may contribute to the diagnosis. This case illustrates the importance of careful evaluation of a patient's past medical history. It also brings to question whether metal skin patch tests should be done routinely preoperatively for individuals with a history of any type of allergies.
